# Mass Spectrometry-Based Analysis of Surface Proteins in *Staphylococcus aureus* Clinical Strains: Identification of Promising k‑mer Targets for Diagnostics

**DOI:** 10.1021/acs.jproteome.5c00321

**Published:** 2025-08-07

**Authors:** Ema Svetlicic, Leonarda A. Alarcon, Roger Karlsson, Carsten Jers, Ivan Mijakovic

**Affiliations:** † Novo Nordisk Foundation Center for Biosustainability, 5205 Technical University of Denmark, 2800 Kgs. Lyngby, Denmark; ‡ Clinical Microbiology, 378993 Sahlgrenska University Hospital, Region Västra Götaland, 40530 Gothenburg, Sweden; § Department of Infectious Diseases, Sahlgrenska Academy, 3570 University of Gothenburg, 40530 Gothenburg, Sweden; ∥ Nanoxis Consulting AB, 40016 Gothenburg, Sweden; ⊥ Department of Biology and Biological Engineering, Division of Systems and Synthetic Biology, Chalmers University of Technology, 412 96 Gothenburg, Sweden

**Keywords:** tryptic shaving, Staphylococcus aureus, surface proteins, proteomics, diagnostic peptides

## Abstract

Surface proteins of Gram-positive bacteria are critical for adherence to host tissues, evasion of the immune system, and interaction with the environment. They can be utilized as biomarkers in diagnostics, for vaccine development, and as therapeutic targets due to their accessibility and role in pathogenicity. If utilized as diagnostic targets, surface biomarkers should be highly conserved across different strains of the pathogen, unique to the species to avoid cross-reactivity, abundantly expressed on the bacterial surface, and accessible to antibodies or detection reagents. Mass spectrometry-based proteomics methods have advanced the studies of surface proteins, often in combination with selective enrichment strategies such as tryptic “shaving”. In this study, 11 clinical strains of *Staphylococcus aureus* underwent tryptic shaving to identify common surface proteins. Further bioinformatics analysis confirmed that these proteins are encoded in the core genome of *S. aureus* strains and contain species-specific peptides. In silico analysis identified 26 k-mer peptides in 15 surface proteins with structural accessibility to detection agents, making them the ideal targets for molecular diagnostics or as linear epitope targets for vaccine development or therapeutics. Among the identified candidates were known virulence-associated proteins such as PbpA, Sbi, and Asp23—previously studied in the context of vaccines—as well as uncharacterized proteins encoded by the gene loci SAUSA300_1904 and SAUSA300_1685, whose unique and surface-exposed features suggest unexplored diagnostic potential.

## Graphical abstract

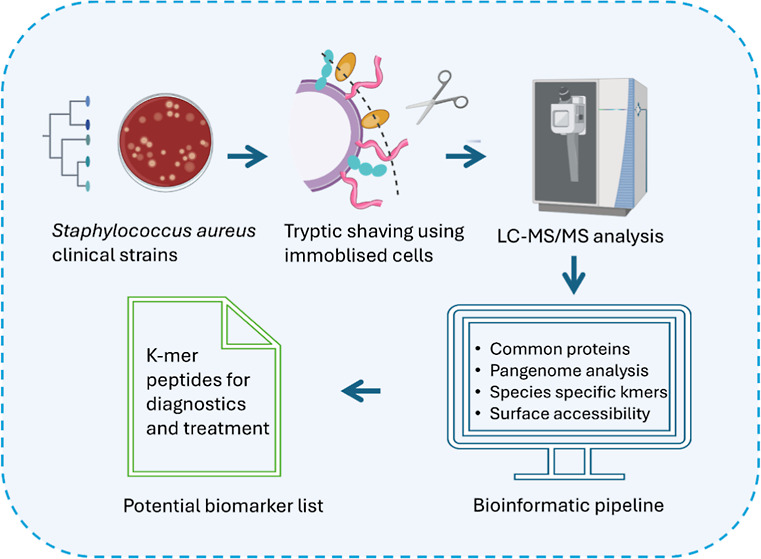

## Introduction

*Staphylococcus aureus* is a Gram-positive bacterium which can cause invasive, potentially life-threatening infections such as sepsis, endocarditis, and pneumonia. The treatment of invasive infections has been reported as extremely challenging due to the pathogens evasion of the host immune system in combination with growing antimicrobial resistance.^1^ The infection mechanism involves a wide range of protein virulence factors, many of which are either secreted into the surrounding environment or attached to the cell wall, in which case they are referred to as surface proteins.^2^ Surface proteins, or more specifically cell wall proteins, have been particularly important in potential clinical applications due to their accessibility, structural and functional diversity, and antigenicity properties.^3,4^ Therefore, these proteins can be utilized as targets for detection of pathogens from clinical and food samples^5–8^ and as targets for vaccine development.^3,9,10^ The known surface proteins of Gram-positive bacteria are usually characterized by sorting signal sequences such as LPXTG and transmembrane domains consisting of hydrophobic stretches. Identification of surface-associated proteins and their exposed epitopes represents a major challenge due to their low abundance and inherent hydrophobicity.^11^ Mass spectrometry-based proteomics techniques have greatly improved their identification through the application of specific sample preparation protocols such as biotinylation and tryptic “shaving”.^12^ The biotinylation approach has been applied in several studies which identified surface proteins in both Gram-negative and Gram-positive bacteria in diverse conditions.^13–17^ However, it was observed that biotin exhibits limited affinity for surface proteins attached via a sortase, resulting in decreased detection sensitivity for these relevant protein antigens.^18^ Tryptic shaving, commonly referred to as surface shaving, is a process in which live bacteria are incubated with the protease trypsin, which results in digestion of ideally only surface-exposed proteins and release of their peptides.^19^ The tryptic peptides are subsequently separated from the cells by centrifugation or filtration and analyzed by mass spectrometry. The method is relatively easy to perform and can also provide information on protein topology since only exposed residues can be accessed by trypsin. However, the drawback of the method is concerns about cell lysis during sample preparation that leads to the release of the cytoplasmic content, resulting in proteins falsely being identified as extracellular.^19^ Notably, surface shaving was more successful in Gram-positive bacteria due to a thicker peptidoglycan layer and higher resistance to lysis.^13^ To avoid the loss of membrane integrity, several strategies have been developed to achieve a controlled digestion using either immobilized trypsin, where the cell suspension is passed through a column containing trypsin^20^ or by cell immobilization, in which case the trypsin solution flows through a channel with cells attached.^21–23^ In addition to mass spectrometry-based identification of surface proteins, various prediction algorithms have been developed for the recognition of the sequence patterns in the proteome, which help to determine the subcellular locations of bacterial proteins. To name a few, pSort^24^ and CELLO^25^ utilize sequence information and training data sets to categorize localization of proteins, TMHMM^26^ detects transmembrane domains in the sequence, and SignalP detects signal sequences which indicate a secretion,^27^ while tools such as SecretomeP^28^ predict nonclassical protein secretion. Extensive explanation and overview of subcellular localization tools can be found elsewhere.^29^ Several studies, however, reported differences between predicted and observed localization, most notably in the proteins predicted as cytoplasmic, which were observed extracellularly, possibly due to nonclassical excretion, translocation mechanisms, and moonlighting properties.^30^ Therefore, the prediction algorithms should be used interchangeably with experimental methods, and proteins predicted as cytoplasmic should not be automatically disregarded as potential diagnostic or vaccine targets. For surface proteins to serve as effective biomarkers, the target proteins must be conserved within the species and should not exhibit homology to host proteins or to proteins from other microorganisms present in the sample. Rather than using alignment methods, which can be computationally consuming, new approaches based on short substrings of DNA or peptide sequences called k-mers were developed in the past decade. By analyzing the frequency and distribution of k-mers across genomes or metagenomic data sets, researchers were able to develop tools such as Kraken^31^ and CLARK^32^ which are now widely used for taxonomic classification of sequenced reads. A recently published paper by Mouratidis et al. identified the shortest set of peptide k-mers that are found in only one species and are absent in every other assembled reference proteome in RefSeq, termed quasi-prime peptides.^33^ The study identified quasi-primes for several pathogens, including *S. aureus*, and highlighted the potential utilization as biomarkers. The quasi-prime peptides were made available through a new database KmerDB.^34^ Peptides represent shorter, defined sequences compared to full proteins, which can contain multiple epitopes. Raising antibodies against peptides allows for more precise targeting of specific linear epitopes within the protein sequence, minimizing the risk of cross-reactivity with other regions of the protein.^35^ This study aimed to identify peptide candidates as potential diagnostic targets by employing an integrated approach that combined tryptic shaving, pangenomics, KmerDB analysis, and structural accessibility assessment. Through this methodology, several promising peptide candidates were identified that demonstrate potential for use in diagnostic applications. The schematic pipeline of this study is depicted in Figure 1.

**Figure 1:**
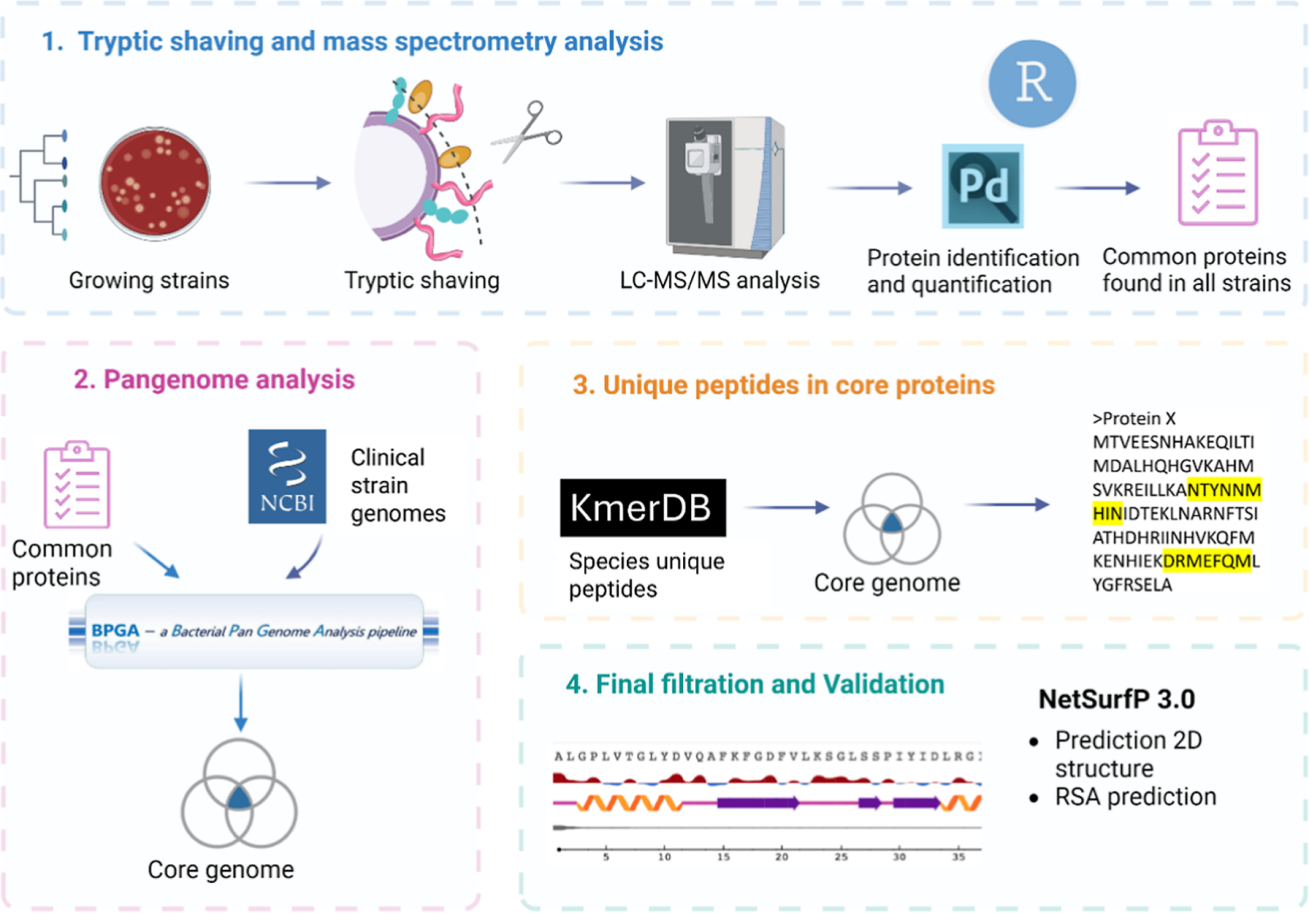
Biomarker discovery pipeline consisting of four main phases. The first phase is the generation of surface-exposed peptides using tryptic shaving of several evolutionarily distinct bacterial strains. The peptides are analyzed by LC–MS/MS and identified by proteomic specialized software. Phase 1 concludes with filtering the strains to obtain only proteins common to all analyzed strains. Phase 2 is further filtering the list of common proteins to include only proteins found in the majority of clinical strains. This is achieved by aligning sequences of common proteins to the core genome of NCBI derived strains. Phase 3 includes matching of the unique peptides of the bacterial species (quasi-prime peptides) to the sequences of the core proteins identifying proteins which contain them. Phase 4 consists of determining the secondary structure of target proteins and filtering sequences of quasi-prime peptides, which are determined to be exposed on the surface.

## Materials and Methods

### Bacterial Strains and Cultivation

The following bacterial strains were obtained from the Culture Collection University of Gothenburg (CCUG) (http://www.ccug.se): *S. aureus* strains CCUG 10778, CCUG 15915, CCUG 1799, CCUG 1800T, CCUG 1801, CCUG 2353, CCUG 2354, CCUG 259252, CCUG 41586, CCUG 41879, and CCUG 54581. The strains were cultivated on Blood Agar medium made of Columbia Agar Base plus 5% horse blood at 37 °C, with 5% CO_2_, overnight (18 h). The biomass was collected and suspended in phosphate-buffered saline (PBS). Bacterial cell suspension optical densities (OD) were measured at a wavelength of 600 nm and adjusted in 1 mL of PBS to an OD_600_ of 1.00 (10^9^ cfu/mL). The biomass was washed with PBS three times and resuspended in 150 μL of PBS.

### Surface Shaving of the Selected Strains

Cell suspension in a volume of 150 μL was slowly injected into the LPI HexaLane Flow Cell (Nanoxis Consulting AB, Sweden) using a pipet. The Flow Cell was incubated for 30 min at room temperature to allow for the immobilization of cells on the channel surface. The Flow Cell channels were washed with 200 μL of PBS to remove unbound bacteria. Enzymatic digestion of the surface-exposed proteins was performed by injecting trypsin (20 μg/mL in 20 mM PBS, pH 8.0) into the Flow Cell channels and incubating for 20 min at room temperature. The generated peptides were eluted by injecting 100 μL of PBS buffer (pH 8.0) into the channels. The eluted peptides were collected at the outlet ports, and peptide solutions were incubated at room temperature overnight at 37 °C with the addition of 1 μg of trypsin. The peptides were desalted using SOLAμ cartridges (Thermo Fischer Scientific) as follows: the columns were primed with 100% methanol, equilibrated with 80% acetonitrile/0.1% trifluoracetic acid and washed twice with 3% acetonitrile/0.1% formic acid. The peptides were loaded and washed twice with 3% acetonitrile/0.1% formic acid. Finally, peptides were eluted with 60% acetonitrile/0.1% formic acid, dried, and subsequently frozen at −20 °C until analysis by MS.

### LC–MS/MS Analysis

For each sample, peptides were loaded onto a 2 cm C18 trap column (Thermo Fisher 164946), connected in-line to a 15 cm C18 reverse-phase analytical column (Thermo EasySpray ES904) using 100% buffer A (0.1% FA in water) at 750 bar, using the Thermo EasyLC 1200 HPLC system, and the column oven operating at 45 °C. Peptides were eluted over a 60 min gradient ranging from 10 to 60% of 80% ACN, 0.1% FA at 250 nL/min, and the Exploris 480 instrument (Thermo Fisher Scientific) was run in a DDA-MS2 top10 method. Full MS spectra were collected at a resolution of 70,000, with an AGC target of 3 × 10^6^ or maximum injection time of 20 ms, and a scan range of 300–1750 *m*/*z*. The MS2 spectra were obtained at a resolution of 17,500, with an AGC target value of 1 × 10^6^ or maximum injection time of 60 ms, a normalized collision energy of 25, and an intensity threshold of 1.7 × 10^4^. Dynamic exclusion was set to 60 s, and ions with a charge state <2 or unknown were excluded.

### Proteomics Downstream Analysis

The raw files were analyzed by using Proteome Discoverer 2.5. The spectra were matched against the Uniprot database of *S. aureus* strain USA300 (UP000001939).

The strain is a community associated methicillin-resistant *S. aureus* frequently isolated from patients; thus, it was chosen as a reference proteome. Dynamic modifications were set as oxidation (M) and acetyl on protein N-termini. All results were filtered to a 1% FDR. The peptide table was further processed in R Studio using a custom-made script employing the packages tidyr, dplyr, ggplot2, corrplot, reshape2. The processing included calculating iBAQ values for each protein using the formula: (sum of peptide intensity intensity)/(theoretical number of peptides). The resulting protein table was then filtered to exclude proteins not quantified in any of the samples. Moreover, if a protein appeared in only one out of three replicates, it was filtered out. Finally, proteins that were detected in all 11 strains were kept as well as the proteins represented with more than 2 peptide-to-spectrum matches (PSMs).

### Cellular Localization Prediction

The FASTA sequence of the proteome of *S. aureus* USA 300 (UniProt ID UP000001939) was searched with Secretome P1.0, pSort v3.0.2, and SignalP 4.0 web tools with settings for Gram-positive bacteria.

### Pangenome Analysis

Genome sequences of *S. aureus* strains were downloaded from the National Center for Biotechnology Information (NCBI) database. The isolates were filtered using the Pathogen Detection browser (https://www.ncbi.nlm.nih.gov/pathogens/isolates) in order to include complete genomes with contigs which originate from clinical strains isolated from human sources in the last ten years. The filtration resulted in 467 genomes which were reannotated using Prokka v1.14.5.^36^ Pangenome analysis was conducted using the Bacterial Pangenome Analysis Pipeline (BGPA).^37^ Genes were considered core genome genes if they were detected in all strains with 70% homology. The list of core genome genes was used as a database for matching protein sequences from tryptic shaving. The matching was performed using standalone blast+^38^ and proteins found in tryptic shaving were considered part of the core proteome if they exhibited >95% sequence similarity.

### Pathway Enrichment Analysis

To explore the functional context of the identified surface proteins, pathway enrichment analysis was performed using the STRING database.^39^ The protein list containing core *S. aureus* proteins identified by the BPGA pipeline was input into STRING using *S. aureus* USA300 complete proteome. Functional enrichment was assessed for Kyoto Encyclopedia of Genes and Genomes (KEGG) pathways using the built-in enrichment tool within STRING. Enrichment was calculated based on the hypergeometric test, with the whole genome of *S. aureus* serving as the background. *P*-values were adjusted for multiple testing using the Benjamini–Hochberg method to control the false discovery rate (FDR). KEGG pathways with an adjusted *p*-value (FDR) < 0.05 were considered significantly enriched.

### Unique Sequences in the Core Genome

The list of species-specific heptamer peptides termed quasiprime peptides was obtained from a database, kmerDB (https://kmer.pennstatehealth.net/kMerDB/) Heptamer strings were matched to the common core protein FASTA file obtained in pangenome analysis and surface shaving experiments.

### Surface Accessibility of Quasi-Prime Peptides

To assess whether the unique peptides are likely to be accessible to the potential antibodies, the relative solvent accessibility (RSA) and secondary structure of the target proteins were predicted using NetSurfP-3.0.^40^ The aim was to identify the quasi-prime peptides that have seven continuous surface-exposed amino acid residues (RSA > 20%).

## Results

### Tryptic Shaving Experiments Identify 388 Common Proteins in 11 Strains of *S. aureus*

Eleven *S. aureus* strains with fully sequenced genome were subjected to surface shaving with immobilized cells using the lipid-based protein immobilization (LPI) technology, as described before.^21^ A detailed description of the 11 strains is presented in Table S1. The strains were chosen from the CCUG collection based on the dendrogram shown in Figure S1. In addition, this study employed semiquantitative analysis using the iBAQ (Intensity-Based Absolute Quantification) method. The named quantification approach enables the estimation of protein abundance based on the sum of the intensity of peptides identified by mass spectrometry divided by the number of theoretical peptides of the protein.^41^ A total of 873 proteins were identified across all of the samples (Supporting Information Table_S2.xlsx). The number of proteins detected varied depending on the strain. For example, strain CCUG2354 had on average 825.6 identified proteins, while in strain CCUG15915 there were on average 590.6 proteins identified. Generally, the biological replicates had a similar number of identified proteins (Figure 2A), indicating the reproducibility of the surface-shaving procedure. However, the differences in the proteins identified across the *S. aureus* strains suggest a variability in the composition of their surface proteomes. Moreover, a binary heatmap visualizing present/absent proteins showed clear clustering of biological replicates and variation in identified proteins among different strains, which clustered into three distinct clades (Figure 2B).

**Figure 2:**
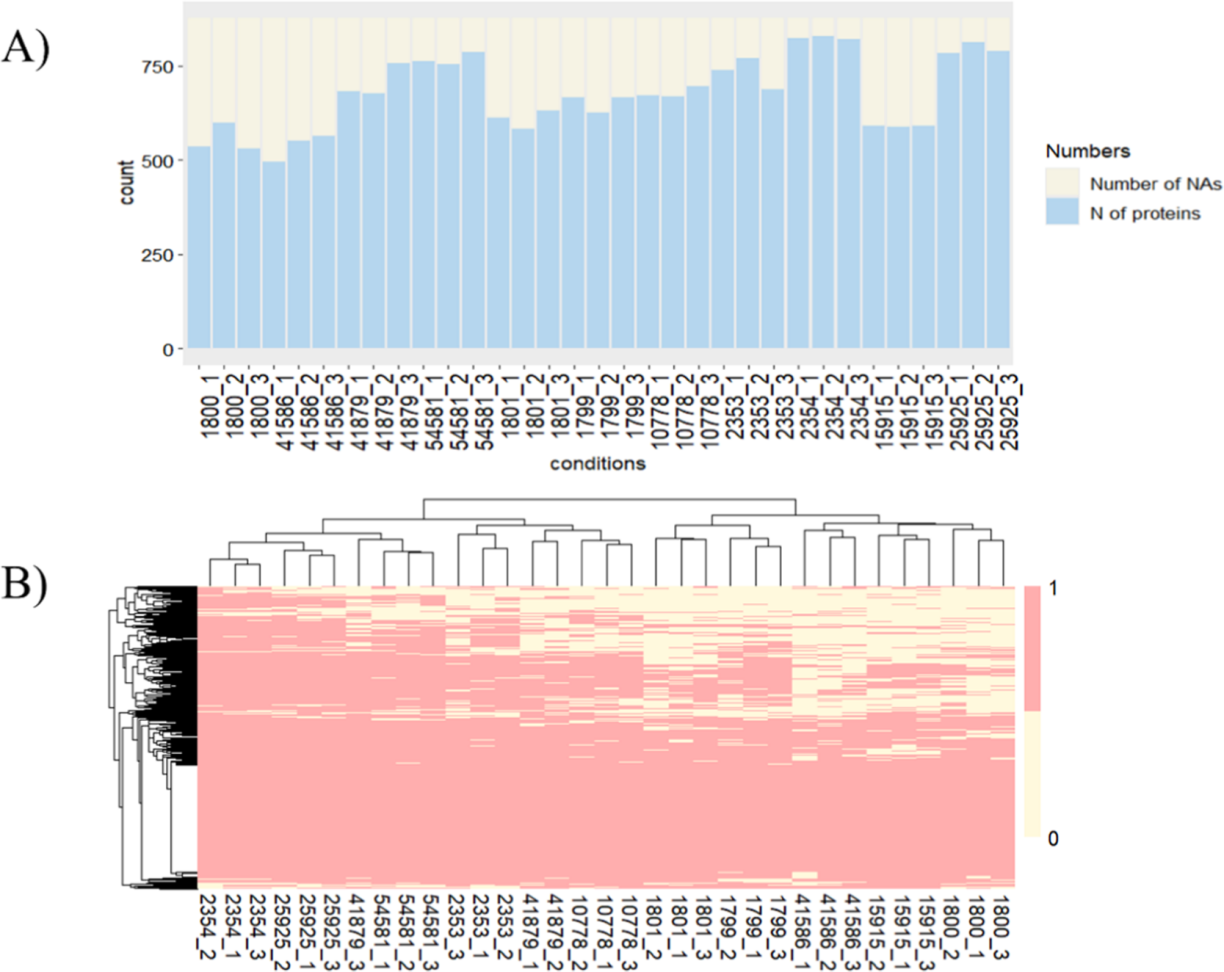
Results of the tryptic shaving experiment of 11 *S. aureus* strains analyzed in biological triplicates. (A) Number of identified proteins for individual samples. (B) Two-way hierarchical clustering heatmap visualizing present/absent proteins across samples.

To determine which proteins are common to all strains, proteins detected in less than two biological replicates were filtered out, and finally only proteins quantified in all 11 strains were kept. Furthermore, proteins with only one peptide to spectrum match (PSMs) were removed, resulting in 388 common proteins. The prediction algorithm pSort was employed for prediction of protein localization for proteins in *S. aureus* USA300 proteome, in protein sequences of all proteins identified (*N* = 873), as well as proteins identified in all strains (*N* = 388) (Figure S2A). The highest number of proteins in all categories belonged to cytoplasmic proteins. Even among proteins identified in the surface shaving experiments, the algorithm predicted >70% cytoplasmic proteins. Along with pSort, the tools signalP and SecretomeP were employed to identify a signal peptides and nonclassically secreted peptides, respectively (Figure S2B). The nonclassically secreted peptides are peptides which are termed secreted but do not contain signal peptide or any apparent amino acid pattern. The proteins termed secreted in the common proteins were then compared among the named prediction tools, and pSort and signalP overlapped in 15 proteins, with SignalP predicting more than twice as many secreted or on the cell surface. Both pSort and SignalP, however, did not identify nonclassically secreted proteins, whereas SecretomeP identified the highest number of secreted proteins, with 74 being nonclassically secreted (Figure S2C). While cell lysis cannot be excluded and probably happened to some extent due to tryptic digestion of membrane proteins, the proteins labeled as cytoplasmic were not excluded from further analysis, as they were identified in all strains and could potentially be present on the surface. In addition to qualitative assessment of surface proteins in the analyzed strains, iBAQ was employed to estimate a semiabsolute amount of protein within the sample. The distribution of protein amounts (iBAQ values) across all samples is shown in a boxplot in Figure 3A. Next, an assessment of the most abundant proteins was made by calculating the mean of the abundances through all groups. The ten most abundant proteins based on total iBAQ mean across all strains were immunoglobulin G-binding protein A (Spa, A0A0H2XJH7), acyl carrier protein (AcP), 30S ribosomal proteins (RpsC, RpsL, and RpsE), UPF0337 domain-containing protein (Q2FIG2), secretory antigen (SsaA), phage protein (A0A0H2XG38), UPF0478 protein (Q2FFZ9), and elongation factor Tu (Tuf) (Figure 3B).

**Figure 3:**
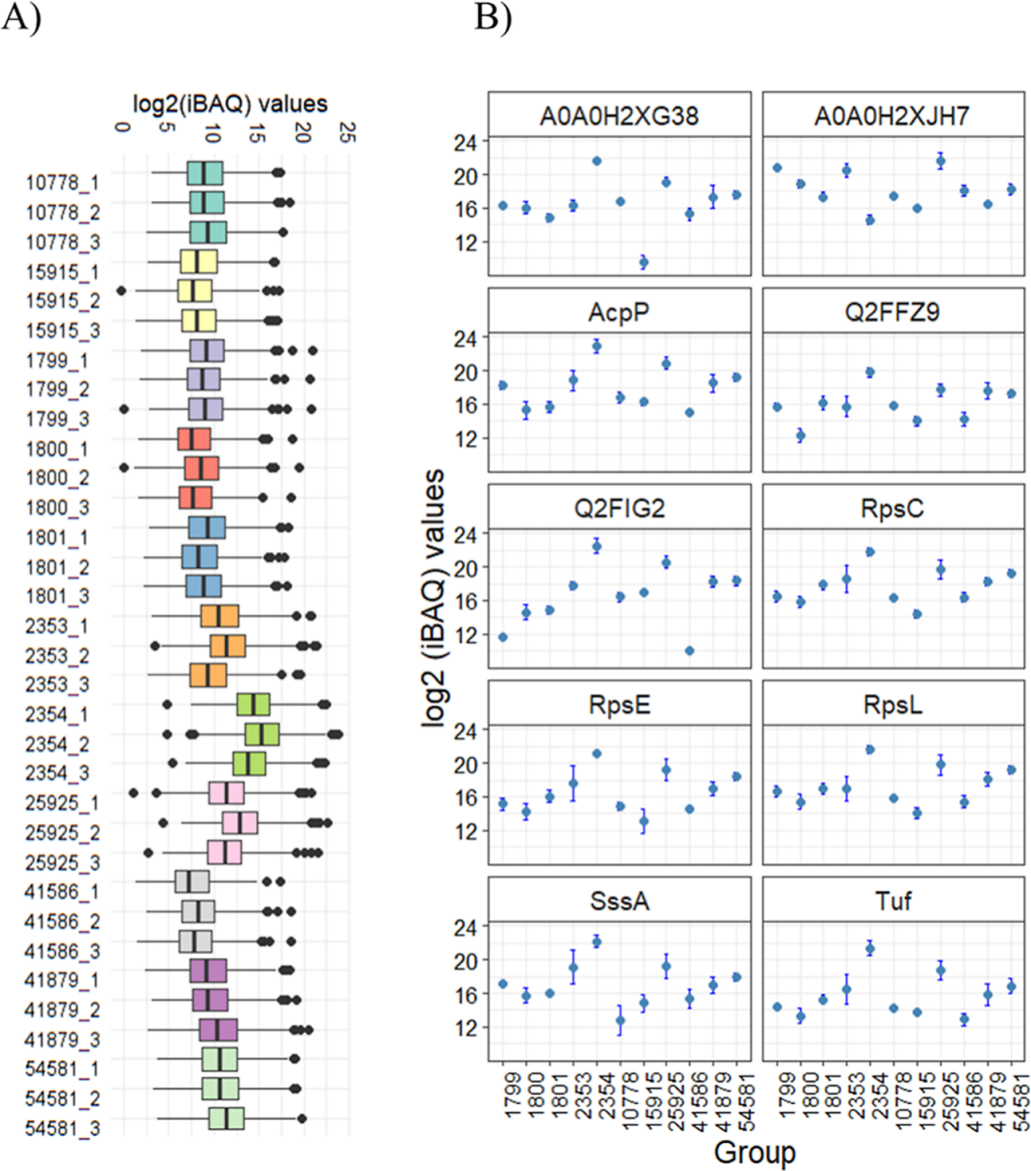
Quantitative overview of proteins identified by tryptic shaving across samples. (A) Boxplots depicting log_2_(iBAQ) values across all samples (B) log_2_(iBAQ) intensities of the 10 most abundant proteins depicted as mean values with standard deviation in the various strains.

### Identification of Surface Proteins Conserved in *S. aureus* Full Genome Sequences

To verify conservation in a greater number of *S. aureus* strains, the proteins identified through tryptic shaving in all strains were compared to a core genome generated from several hundred *S. aureus* clinical strains, with genome sequences obtained from NCBI. The strains included in the analysis yielded a pangenome size of 5363 proteins. The BPGA pipeline used in the analysis classifies proteins into core proteins, which are present in all strains, unique proteins, which are present in only one strain, and the remaining proteins, which are categorized as accessory proteins. The rarefaction curve of core proteins shows that the core proteome, after an initial sharp drop, plateaued and exhibited a minimal decrease upon addition of more genomes (Figure 4A). The core proteome consisted of 1806 proteins; the accessory proteome had 2862 proteins, and there were 695 unique proteins (Figure 4B). The common proteins from the surface shaving experiment were matched by blastp+ algorithm against the core proteome to find the overlap between the experimental data and a broader core proteome data set. Of the experimentally identified 388 common proteins, there were 346 proteins with above 95% similarity to proteins in the core genome. These 346 proteins were designated as common core proteins, as they were identified through tryptic shaving in all strains and were also part of the *S. aureus* core genome. The full list of 346 common core proteins is shown in Supporting Information Table_S3.xlsx. Pathway enrichment analysis revealed that common core proteins were enriched in pathways related to carbon metabolism (e.g., glycolysis, the TCA cycle, and pyruvate metabolism) and processes related to ribosome function (Figure S3).

**Figure 4:**
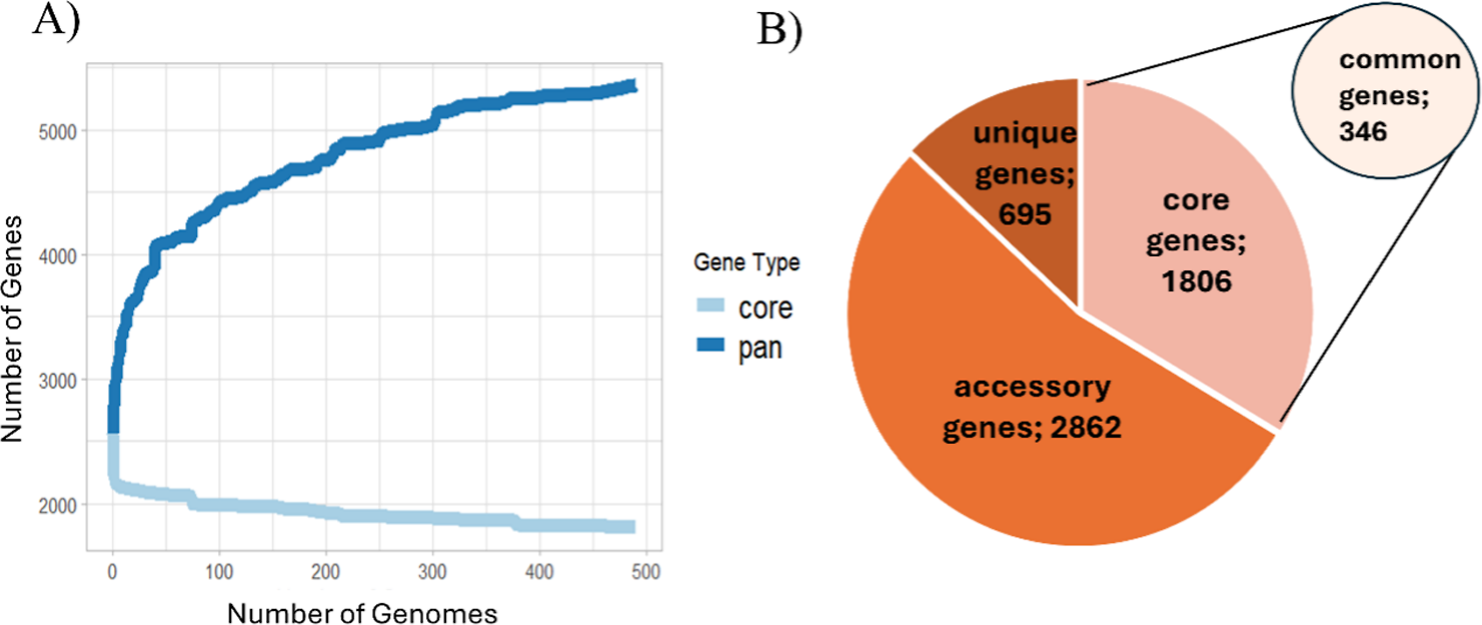
Pangenome analysis of clinical strains of *S. aureus* whose proteome sequence was obtained from NCBI Pathogen Detection resources. (A) Rarefaction curve shows plateauing of the core genome. (B) Results of the pangenome analysis in terms of number of core, accessory, and unique genes, where core genes represent the genes found in all tested strains.

### Quasi-Prime Peptide Mapping Identifies 26 Peptide Targets in 15 Proteins

Potential diagnostic biomarkers should be specific to the targeted species and absent from other species. The proteins that were identified in tryptic shaving and pangenome analysis as core proteins can have an overall low homology to other species or have stretches of amino acids which are species-specific. As previously mentioned, KmerDB, which contains more than 5000 heptamer k-mer sequences unique to *S. aureus* (quasi-prime peptides)^34^ was used to identify species-specific protein fragments in the common core proteins. The heptamer sequences were downloaded from KmerDB and string-matched to the fasta sequences of the 346 common core proteins. In total, 285 heptamers were matched to 132 proteins in the common core proteins (Supporting Information Table_S4.xlsx). These unique stretches of amino acids must also be exposed on the surface of the protein to ensure that they are accessible to antibodies or other detection agents. The amino acid surface exposure measured by RSA was calculated for all 132 proteins using a sequence-based tool NetSurfP 3 (Supporting Information Table_S5.xlsx). The residues having RSA < 0.25 were filtered out from the data set. The data was further filtered to include only peptides with seven consecutive surface exposed residues. For example, a heptamer FNPENTK in signal peptidase was predicted to have all RSA values above 0.25, therefore it was deemed a suitable candidate. However, a heptamer QCGHHLN in formate acetyltransferase only had the first two residues exposed on the surface; therefore, the named peptide was eliminated (Figure 5A). This criterion greatly narrowed down the possible biomarker candidates, leaving 26 surface-exposed heptamer peptides from 15 proteins. The 26 k-mers are shown in Table 1. Since the quasi-prime peptides in KmerDB were identified only from reference genomes of each species, the 26 potential peptide targets were searched against 467 *S. aureus* proteome sequences included in the pangenome analysis. The majority of k-mers (23/26) were found in over 99% of the strains, while three k-mers were present at lower frequencies: KHNFNPE in 74.3% yield, NIVNHHQ in 77.9% yield, and YIDYYKH in 98.7% yield of the *S. aureus* strains (Table 1).

**Figure 5:**
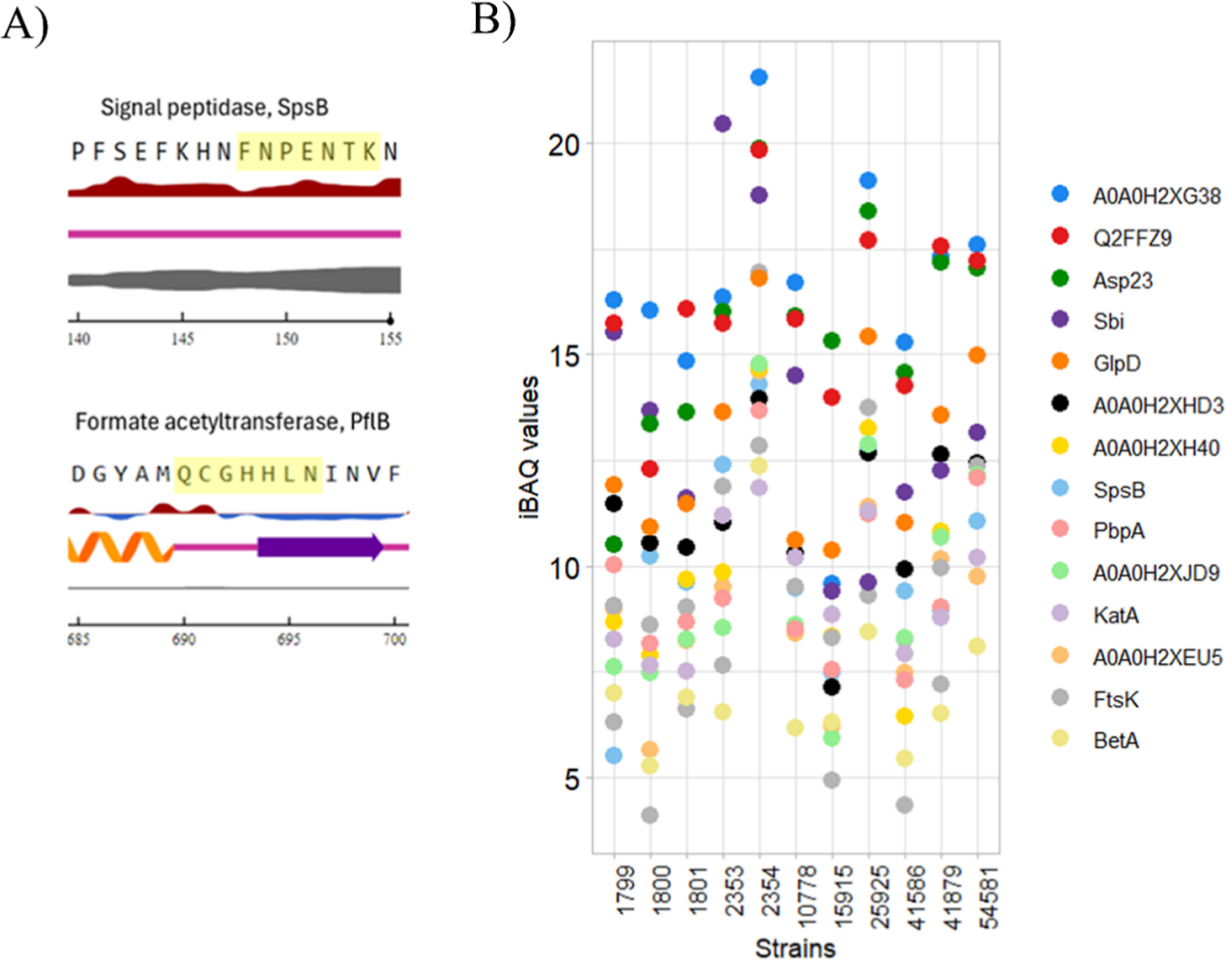
Relative surface accessibility (RSA) of quasi-prime peptides. (A) Results of the NetSurfP 3 for the representative two proteins. The first line shows a protein sequence with the quasi-prime peptide sequence highlighted in yellow. The second line represents the RSA values, where red indicates an exposed residue and blue a buried one. The third line shows the predicted secondary structure of the protein region, either a coil in pink, a helix in orange, or a beta-sheet in blue. Finally, the fourth line shows the probability of the region being disordered, where the thicker line indicates higher probability. (B) Protein abundance measured as log_2_(iBAQ) values of the proteins that contain quasi-prime peptides with 7 consecutive exposed residues.

**Table 1:** Quasi-Prime Peptides, Comprised of Seven Unique Amino Acids (K-mers) Specific to *S. aureus*, Identified within the Core Proteome Established in This Study^a^

gene name/locus	uniprot accession	protein name	K-mers	start	end	appears in % strains
*spsB*	A0A0H2XEA7	signal peptidase I	KHNFNPE	145	151	74.3
SAUSA300_0508	A0A0H2XEU5	excinuclease ABC subunit B	CAEGHHP	6	12	91.9
			HHPWNQA	10	16	91.9
			MVCQTCA	1	7	99.4
SAUSA300_1904	A0A0H2XG38	phage protein	CVFKFVF	7	13	99.8
			FIKCVFK	4	10	99.8
			MWNFIKC	1	7	99.8
			WNFIKCV	2	8	99.8
SAUSA300_1304	A0A0H2XH40	uncharacterized protein	ETEMTMP	204	210	99.8
SAUSA300_1729	A0A0H2XHD3	NERD domain-containing protein	QEDYNHM	73	79	99.6
*ftsK*	A0A0H2XHM5	DNA translocase FtsK	NIVNHHQ	241	247	77.9
*ftsA*	A0A0H2XHQ1	cell division protein FtsA	HEHVQDK	446	452	99.8
			HQEHKQN	439	445	100
SAUSA300_1003	A0A0H2XJD9	uncharacterized protein	MVAPQYY	155	161	99.6
*pbpA*	A0A0H2XJZ5	penicillin-binding protein 1	LRISYIM	31	37	99.8
*betA*	Q2FDP9	oxygen-dependent choline dehydrogenase (CDH)	YIDYYKH	546	552	98.7
			YKHGVHD	550	556	100
*sbi*	Q2FE79	immunoglobulin-binding protein Sbi	DNKAPHD	166	172	99.6
			QDNKAPH	165	171	99.6
*asp23*	Q2FEV0	alkaline shock protein 23	NQEPQFK	33	39	99.8
			VMTQKEW	144	150	99.8
SAUSA300_1685	Q2FFZ9	UPF0478 protein	DKWQNRH	130	136	100
			KWQNRHY	131	137	100
*katA*	Q2FH99	catalase (EC 1.11.1.6)	YNQRQDD	417	423	99.8
*glpD*	Q2FHD8	aerobic glycerol-3-phosphate dehydrogenase GAPDH	AQHGNNQ	546	552	100
			QTSQYHD	463	469	100

a The proteins are represented by their common name, gene locus and Uniprot Accession number of the *S. aureus* USA300 strain.

To determine the relative abundance of the 15 target proteins within the tryptic shaving samples, the average log_2_(iBAQ) values were plotted for each strain (Figure 5B). On average, the most abundant protein biomarker candidates within the samples seemed to be phage protein, alkaline shock protein 23 Asp23, immunoglobulin-binding protein Sbi, UPF0478 protein, and aerobic glycerol-3-phosphate dehydrogenase GAPDH.

## Discussion and Conclusions

The present study sets out to identify surface proteins that are conserved in and unique to *S. aureus* as potential biomarkers for diagnostics. The number of identified proteins is higher than in the previous research of *S. aureus* surfaceome,^20,42^ which might be attributed to differences in growth conditions, cell lysis, mass spectrometry analysis, and proteomics software used. Nevertheless, the results are comparable, as the study also shows heterogeneous protein expression between different *S. aureus* strains and, more importantly, an overlap between identified proteins. The proteins overlapping between the two studies, among others, include probable malate/quinone oxidoreductase, 30S and 50S ribosomal proteins, fructose-bisphosphate aldolase FbA, pyruvate dehydrogenase complexes E1 (PhdAB), elongation factors Tu (EF-Tu) and G (FusA), enolase (Eno), bifunctional purine biosynthesis protein PurH, pyruvate dehydrogenase PdhAB, and glyceraldehyde 3-phosphate dehydrogenase GAPDH. These proteins are considered intracellular; however, in several of the named proteins, moonlighting properties were observed in *S. aureus* as well as in other bacteria. The glycolytic enzyme GAPDH was among the first identified moonlighting proteins, with a discovered role in iron scavenging during infection when iron is limited, thereby playing a crucial role in pathogenesis.^43^ Moreover, it was found that EF-Tu moonlights on the surface and binds to host proteins.^44^ Eno and FbA were also revealed to be exported to the cell surface under various growth conditions in different species.^45,46^ In fact, excretion of cytoplasmic proteins appears to be a common physiological feature in *S. aureus* as well as other bacteria.^47,48^ Computational prediction tools such as pSORT, also used in this study, localize these proteins only in the cytoplasm. The reason for the discrepancy is, as previously noted, poor understanding of these mechanisms and lack of any apparent recognition patterns.^47^ Therefore, the mass spectrometry-based analysis of surface proteins employed in this study provides valuable insights into surface-associated proteins. Nevertheless, cell lysis during sample preparation cannot be excluded, and the portion of proteins identified could be the result of trypsin penetrating the cytoplasmic membrane and causing the lysis. Therefore, the protein abundance was measured with a semiquantitative approach using iBAQ. The most abundant protein was SpA, a well characterized, known surface-anchored protein which binds to immunoglobulin G, thus hindering the immune response of the host.^49^ While it was detected in all samples subjected to tryptic shaving, the protein was not part of the core genome generated in silico by pangenome analysis. The SpA was previously found in approximately 90% of isolates^50^ and in our study it was part of the accessory genome, and thus excluded from further analysis. Another highly abundant protein was the acyl carrier protein ACP, a protein involved in fatty acid and polyketide syntheses, not previously reported to be surface-anchored. Part of the most abundant proteins found in this study were ribosomal proteins, more specifically, 30S ribosomal proteins. Although known as intracellular proteins having a crucial role in protein synthesis, they have been identified in the secretome, associated with extracellular vesicles, and identified in surfaceome profiling experiment.^51–53^ While it is known that many ribosomal proteins are involved in other functions, the moonlighting properties have not been observed on the surface of bacteria and their exact translocation (if any) is not known.^53^ The discussed proteins were all part of the 388 common proteins identified by tryptic shaving and MS analysis in all 11 strains in at least two biological replicates. As identified in previous studies, the majority of the *S. aureus* core genome includes housekeeping proteins, which are responsible for transcription, translation, RNA processing and modification, and metabolism.^54^ These pathways were also enriched in the common core proteins in this study. Most of the common proteins identified by tryptic shaving were part of the core genome (89%); however, the remaining proteins identified in the surface shaving were filtered out of the further processing, as the study focuses on finding new diagnostic markers, and it is favored that they are present in all of the strains. Next, the proteins were checked for their uniqueness. Instead of using alignment-based algorithms such as BLAST, which can be computationally intensive, this study utilized an already established set of unique peptides (k-mers) present in only *S. aureus*. The usage of k-mers for taxonomic classification is widely used in the annotation of sequence reads in metagenomic research due to their speed and comparable sensitivity.^55,56^ The unique k-mers for *S. aureus* were detected in the previous study by analyzing proteomes from 21,875 species and identifying the unique k-mers of 7 amino acids (heptamers).^33^ By matching those quasi-prime peptides to the core proteins identified by tryptic shaving in this study, we identified 132 proteins that contained a total of 285 unique heptamers. For the application of these proteins in healthcare, such as diagnostics, it is critical that these unique heptamers are exposed on the surface of the protein, in order to be targeted by a receptor or a drug. A commonly used measure of surface exposure is RSA which was calculated for all of the 132 proteins. While the majority of residues had RSA values above the threshold (25%), indicating surface exposure, only 15 proteins contained quasi-prime peptides, with all seven residues predicted as exposed. Some of these proteins, such as PbpA and Sbi, have already been targeted in the vaccine research.^57,58^ In addition, Sbi was one of the most abundant proteins in all samples, confirming its potential in clinical applications. Another highly abundant protein was Asp23, a stress response factor previously identified as a T cell antigen,^59^ and the moonlighting protein GAPDH. The proteins, phage protein (SAUSA300_1904) and UPF0478 protein (SAUSA300_1685), have not been experimentally characterized so far, and not much is known about their properties or function.

A previous study employing surface shaving and proteomic analysis^42^ identified several immunodominant and surface-exposed proteins of *S. aureus*, including clumping factor B (ClfB), iron-regulated surface determinant protein B (IsdB), elastin-binding protein (EbpS), lipoteichoic acid synthase (LtaS), major autolysin (Atl), immunodominant staphylococcal antigen A (IsaA), Sbi, and the cytoplasmic proteins GAPDH and Eno as potential candidates for vaccine or immunotherapy development. The findings in this study align with and extend these observations: Sbi and GAPDH were consistently abundant and surface-accessible across all clinical strains examined. While in this study some of the well-studied immunodominant proteins, such as IsdB and ClfB, were not shortlisted as biomarker candidates, several previously uncharacterized or less-studied proteins—such as those encoded by SAUSA300_1904 and SAUSA300_1685—were identified as surface-exposed, conserved, and species-specific, highlighting their potential as novel diagnostic or therapeutic targets.

These biomarkers, being surface-exposed and unique to the pathogen, can be utilized in the creation of rapid assays that can identify the presence of the bacteria in clinical samples, improving early diagnosis and treatment. Characteristics of the ideal diagnostic test, especially in the developing world, were defined almost two decades ago. The tests should be affordable, sensitive, specific, rapid, equipment-free, and accessible to those who need them.^60^ Incorporation of biomarkers into rapid diagnostic devices using biorecognition elements such as antibodies, bacteriophages, aptamers, and antimicrobial peptides are continuously developed,^61–65^ with some already available on the market.^66^ For the biomarkers to be utilized in diagnostics, an extensive experimental screening of the identified biomarkers should take place to evaluate the biorecognition capabilities of receptors as well as their sensitivity and specificity to *S. aureus*. Since the study uncovers biomarker potential in several cytoplasmic proteins, future studies will aim to experimentally validate the surface localization of these candidate proteins using microscopy-based approaches such as immunofluorescence to distinguish true surface exposure from potential cytosolic contamination. Some limitations of this study should also be mentioned. While quasi-prime peptides offer short, unique sequences favorable for recognition, sequence variants and sequencing errors can result in false identifications of the unique k-mers.^33,33^ Therefore, it is recommended to rely on several biomarkers, which are shown to improve diagnostic accuracy.^67^ Moreover, future work could also evaluate species-specific peptides in excreted *S. aureus* proteins, especially numerous toxins that this bacterium produces.

In conclusion, the data gathered from these analyses was utilized for the selection of potential diagnostic targets by several bioinformatic approaches (1) pangenome analysis to ensure the high coverage of the target proteins among *S. aureus* strains; (2) specificity to the species by matching species-unique peptides (quasi-prime peptides); and (3) prediction of RSA values for the quasi-prime peptides to select for fully exposed peptides. The bioinformatic analysis uncovered 26 peptides in 15 proteins, which could serve as targets for clinical applications. The results presented in this study can greatly reduce the workload and cost of such a screening. Moreover, the pipeline in the analysis can be applied to other pathogen species and thus aid in future strategies to identify diagnostic targets.

## Supplementary Material











## Data Availability

The mass spectrometry proteomics data have been deposited to the ProteomeXchange Consortium via the PRIDE partner repository with the data set identifier PXD061994.

## Notes

List of *S. aureus* clinical strains used for tryptic shaving, dendrogram of the chosen *S. aureus* strains, in silico predicted localization of identified proteins, and pathway enrichment analysis of common core surface proteins (PDF)
Results of mass spectrometry analysis and Proteome Discoverer search (XLSX)
List of common core proteins, which are proteins common to all strains subjected to surface shaving and to all strains in the core proteome, as identified by pangenome analysis (XLSX)
Quasiprime k-mer peptides identified in common core proteins (XLSX)
Surface accessibility of amino acid residues in k-mer peptides (XLSX)
