# Supplementary material for: Mass Spectrometry-Based Analysis of Surface Proteins in Staphylococcus aureus Clinical Strains: Identification of Promising k‑mer Targets for Diagnostics
Source: J Proteome Res. 2025 Aug 7;24(9):4575–85. doi: 10.1021/acs.jproteome.5c00321 (PMC12418492; doi:10.1021/acs.jproteome.5c00321)
Supplement: Supplementary file 1 [file pr5c00321_si_001.pdf]

Mass spectrometry-based analysis of surface proteins in *Staphylococcus aureus* clinical strains: Identification of promising k-mer targets for diagnostics

Ema Svetlicic[1], Leonarda A. Alarcon[2], Roger Karlsson[2,3,4], Carsten Jers[1] and Ivan Mijakovic[1,5]*

[1]Novo Nordisk Foundation Center for Biosustainability, Technical University of Denmark, , 2800, Kgs. Lyngby, Denmark

[2] Clinical microbiology, Sahlgrenska University Hospital, Region Västra Götaland, 40530, Gothenburg, Sweden

[3] Department of Infectious Diseases, Sahlgrenska Academy, University of Gothenburg, 40530, Gothenburg, Sweden.

[4]Nanoxis Consulting AB, 40016, Gothenburg, Sweden.

[5] Department of Biology and Biological Engineering, Division of Systems and Synthetic Biology, Chalmers University of Technology, 412 96, Gothenburg, Sweden

Keywords: Tryptic shaving, *Staphylococcus aureus*, Surface proteins, Proteomics, Diagnostic peptides

*Correspondance

Ivan Mijakovic

ivan.mijakovic@chalmers.se

Table of Contents

Supplementary Table S1. A list of *S. aureus* clinical strains used for tryptic shaving.........S2

Supplementary Figure S1. Dendrogram of the chosen *S. aureus* strains........................S3

Supplementary Figure S2. *In sillico* predicted localization of identified proteins............S4

Supplementary Figure S3. Pathway enrichment analysis of common core surface proteins..S5

Supplementary Table S2. Results of Mass spectrometry analysis and Proteome Discoverer search (xlsx)

Supplementary Table S3. A list of common core proteins which are proteins common to all strains subjected to surface shaving and to all strains in the core proteome (genome) as identified by pangenome analysis (xlsx)

Supplementary Table S4. Quasiprime k-mer peptides identified in common core proteins (xlsx)

Supplementary Table S5. Surface accessibility of amino acid residues in k-mer peptides (xlsx)

Table S1: *Staphylococcus aureus* strains chosen for tryptic shaving and MS analysis along with the respective details on the strains

N°	Specie	Accession N° Genbank	CCUG code	Assembly status	Other culture collections	Place of origin	Sample type
**1**	S. aureus	GCA_002025145.1	CCUG 10778	Complete	ATCC 6538	London UK	Human lesion
**2**	S. aureus	GCA_001879295.1	CCUG 15915	Contig	ATCC 29213	Rockville USA	Wound
**3**	S. aureus	GCA_900457405.1	CCUG 1799	Contig	NCTC 8531	London UK	Human blood, Osteomyelitis
**4**	S. aureus	GCA_006094915.1	CCUG 1800T	Complete	ATCC 12600	London UK	Pleural fluid
**5**	S. aureus	GCA_900636395.1	CCUG 1801	Complete	NCTC 6131	London UK	clinical
**6**	S. aureus	GCA_900474695.1	CCUG 2353	Complete	NCTC 5663	London UK	Unknown
**7**	S. aureus	GCA_900457855.1	CCUG 2354	Contig	NCTC 7428	London UK	Unknown
**8**	S. aureus	GCA_900458145.1	CCUG 25925	Contig	NCTC 10655	London UK	Human leg abscess
**9**	S. aureus	GCA_003052445.1	CCUG 41586	Contig	ATCC 43300	Kansas USA	Human clinical isolate
**10**	S. aureus	GCA_013427085.1	CCUG 41879	Contig	ATCC 33591	New York USA	Human blood
**11**	S. aureus	GCA_900457575.1	CCUG 54581	Contig	NCTC 10833	Czekia	clinical

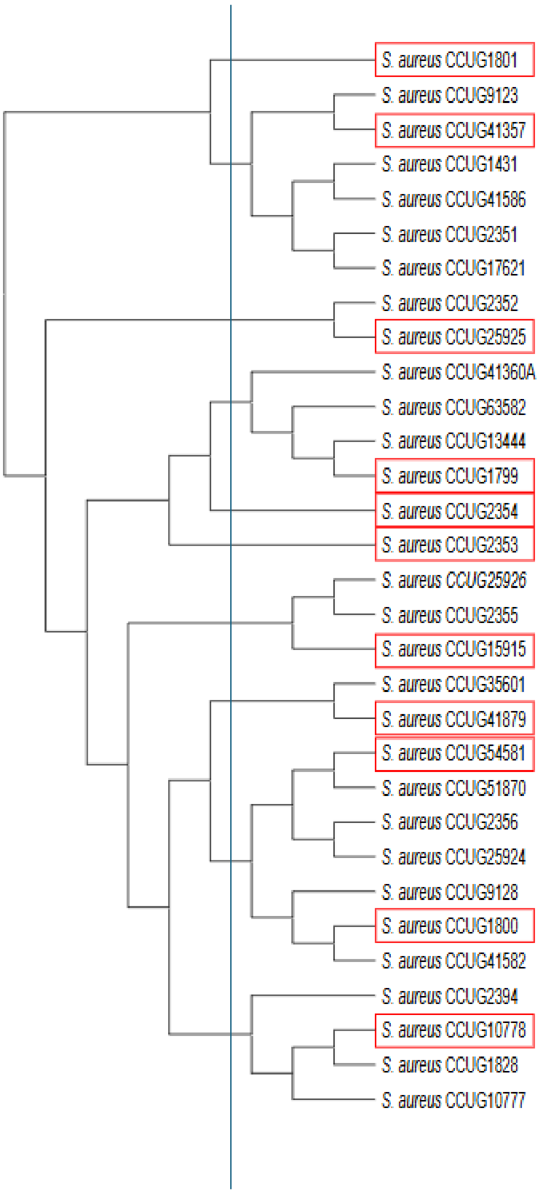

Figure S1: Whole genome dendogram of *Staphylococcus aureus* strains available in the CCUG. The strains chosen for tryptic shaving are circled in red

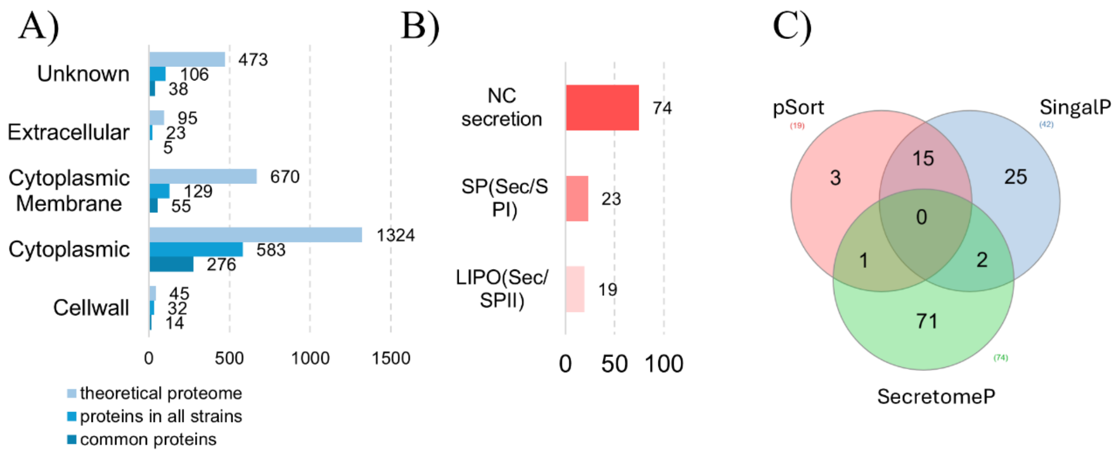

Figure S2: A) Predicted localization of proteins in theoretical *S. aureus* USA300 proteome, of proteins identified in all strains in the tryptic shaving experiment and the subgroup of these proteins found in all strains. B) Predicted localization of common proteins by SignalP and SecretomeP. NC- Non-classical, Sec/SPI: "standard" secretory signal peptides transported by the Sectranslocon and cleaved by Signal Peptidase I, LIPO (Sec/SPII): Lipoprotein signal peptide. C)

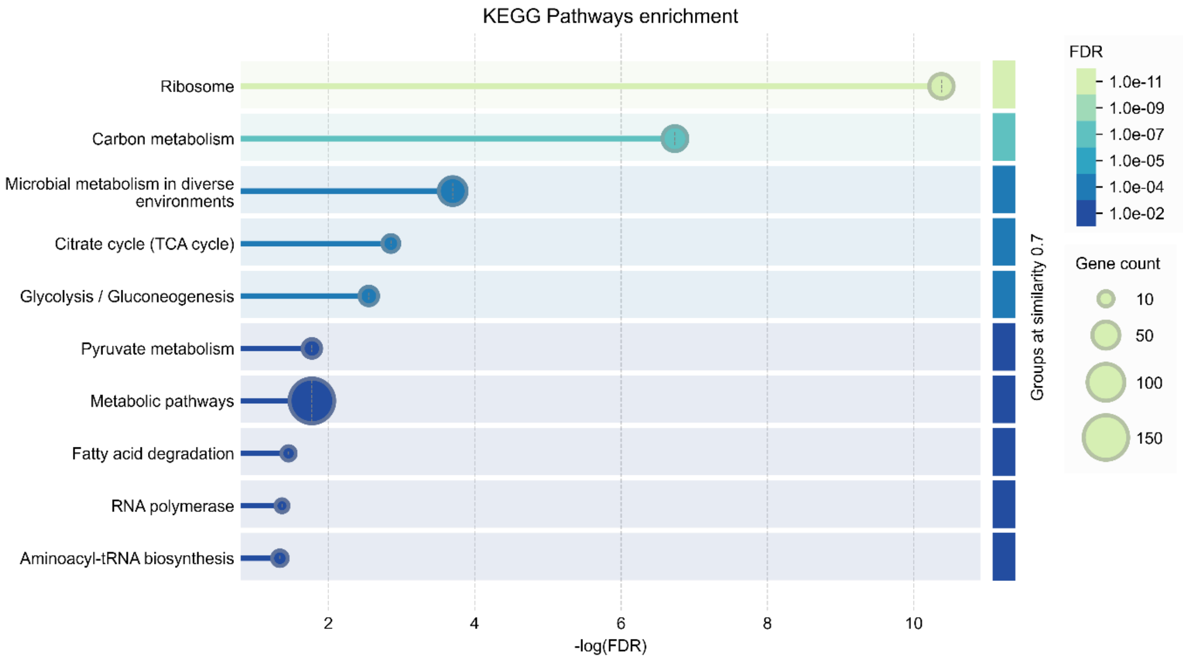

Figure S3: KEGG pathway enrichment analysis of common core surface proteins. The dot plot illustrates the KEGG pathway enrichment of surface-associated proteins, highlighting significantly enriched pathways based on FDR values and gene ratios. Dot size corresponds to the number of genes mapped to each pathway, while color intensity represents the degree of statistical significance.
